# Human Growth Hormone Delivery with a Microneedle Transdermal System: Preclinical Formulation, Stability, Delivery and PK of Therapeutically Relevant Doses

**DOI:** 10.3390/pharmaceutics6020220

**Published:** 2014-05-15

**Authors:** Mahmoud Ameri, Miryam Kadkhodayan, Joe Nguyen, Joseph A. Bravo, Rebeca Su, Kenneth Chan, Ahmad Samiee, Peter E. Daddona

**Affiliations:** Zosano Pharma, 34790 Ardentech Court, Fremont, CA 94555, USA; E-Mails: mameri@zosanopharma.com (M.A.); mkadkhodayan@zosanopharma.com (M.K.); jnguyen@zosanopharma.com (J.N.); jbravo@zosanopharma.com (J.A.B.); rsu@zosanopharma.com (R.S.); kchan@zosanopharma.com (K.C.); asamiee@zosanopharma.com (A.S.)

**Keywords:** human growth hormone, rhGH, stability, transdermal microneedle patch, pharmacokinetics

## Abstract

This study evaluated the feasibility of coating formulated recombinant human growth hormone (rhGH) on a titanium microneedle transdermal delivery system, Zosano Pharma (ZP)-hGH, and assessed preclinical patch delivery performance. Formulation rheology and surface activity were assessed by viscometry and contact angle measurement. rhGH liquid formulation was coated onto titanium microneedles by dip-coating and drying. The stability of coated rhGH was determined by size exclusion chromatography-high performance liquid chromatography (SEC-HPLC). Preclinical delivery and pharmacokinetic studies were conducted in female hairless guinea pigs (HGP) using rhGH coated microneedle patches at 0.5 and 1 mg doses and compared to Norditropin^®^ a commercially approved rhGH subcutaneous injection. Studies demonstrated successful rhGH formulation development and coating on microneedle arrays. The ZP-hGH patches remained stable at 40 °C for six months with no significant change in % aggregates. Pharmacokinetic studies showed that the rhGH-coated microneedle patches, delivered with high efficiency and the doses delivered indicated linearity with average *T*_max_ of 30 min. The absolute bioavailability of the microneedle rhGH patches was similar to subcutaneous Norditropin^®^ injections. These results suggest that ZP-transdermal microneedle patch delivery of rhGH is feasible and may offer an effective and patient-friendly alternative to currently marketed rhGH injectables.

## 1. Introduction

There has been a vast growth in the development of biopharmaceutical products over the past two decades and most of these are administered by intravenous or subcutaneous injection. Chronic diseases, such as osteoporosis, diabetes, and growth hormone deficiency, are treated with therapeutic peptides and proteins and for a drug to be of clinical utility it requires years of good patient compliance to attain its therapeutic effect. Thus, a patient-friendly drug delivery system would be desirable alternative for the patient. There is still a paucity of effective methods for administration of therapeutic proteins to patients. Oral delivery is not a viable delivery method for therapeutic proteins due to poor absorption and degradation in the gastrointestinal tract and liver [1,2]. Several alternative routes that avoid first-pass effects and proteolytic degradation include nasal, pulmonary, and transdermal delivery [3]. The nasal route has a limited surface area, also the fast mucociliary clearance, and drainage into the esophagus can result in highly variable absorption [4]. The pulmonary route offers a large and highly vascularized mucosal surface for drug absorption, but the accessibility with currently available inhalers is limited and can additionally be compounded by variability from patient self-administration [5]. The transdermal route offers a large and varied surface as well as ease of application. There are limitations for this type of delivery since molecules with molecular weight greater than 1 kDa are unable to cross the stratum corneum. However, the use of skin permeation enhancers such as DMSO (dimethyl sulfoxide) may be able to transiently decrease the barrier property of the stratum corneum and allow for drug absorption. Permeation enhancer can cause localized irritation as an adverse side effect [6]. To eliminate the use of enhancers, a number of technologies are being developed that utilize physical/mechanical means to traverse the stratum corneum and deliver the drug. The approaches involve ablation of the skin to create aqueous pathways for peptides and proteins. The energy modalities being evaluated include laser [7], radiofrequency [8], heat [9], and sonophoresis [10]. The ablation treatment is followed by transdermal drug patch application over the ablated skin site. Alternatively, transdermal microneedles have been developed as a pretreatment approach prior to application of a transdermal drug patch [11].

Hollow microneedles with aqueous formulation infusion [12,13] and biodegradable drug-incorporated microneedles [14,15,16,17] have been developed and tested.

**Figure 1:**
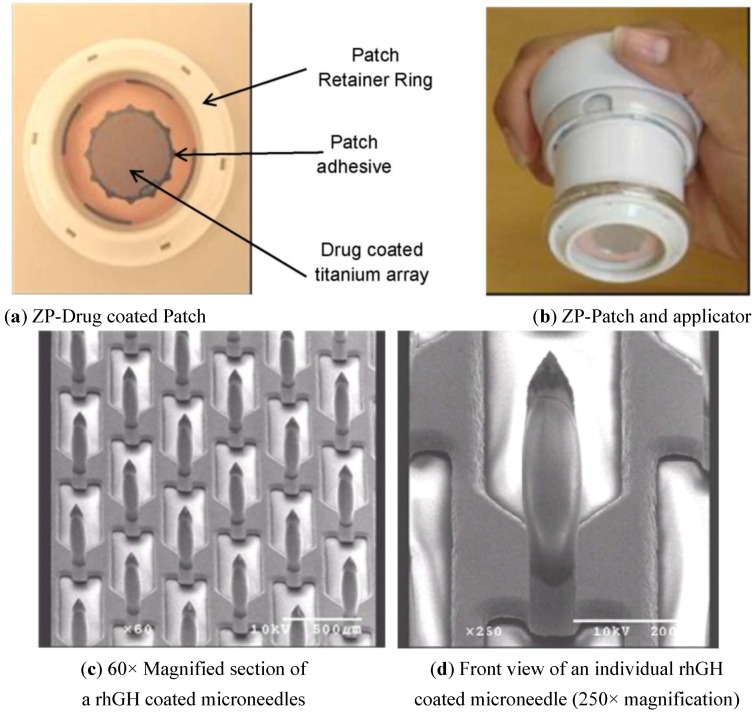
Transdermal microneedle patch delivery system (**a**) 5 cm^2^ adhesive patch with microneedle array (3 cm^2^) in applicator ring; (**b**) Zosano Pharma (ZP)-Patch press fit onto the bottom of the applicator; and applicator; (**c**) 60× magnification of recombinant human growth hormone (rhGH) coated microneedles (580 microneedles/cm^2^, length 340 µm and tip angle 60°), rhGH coated at 0.5 mg/3 cm^2^ array; and (**d**) Front view of an individual rhGH coated microneedle (250× magnification).

Previous articles [8,15,16] that studied rhGH administration via the microneedle route had non-therapeutic dosages. We describe here a novel drug-coated microneedle patch delivery system that targets high therapeutic doses of rhGH to the epidermal/dermal layer for rapid and efficient delivery. The Zosano Pharma (ZP)-patch system consists of a titanium microneedle array attached to an adhesive patch seated in a retainer ring, and an applicator (Figure 1a). The patch in retainer ring is attached to the bottom of the applicator. The applicator (Figure 1b) is actuated through spring force, which breaks the adhesive from the retainer ring and applies the patch onto the skin site. The drug-coated microneedles physically break the stratum corneum and penetrate the epidermis/dermis, where the dry drug coating is dissolved by the surrounding skin interstitial fluid. The ZP-patch system has recently been tested in a Phase 2 clinical study with the delivery of human parathyroid hormone (1–34) peptide for the treatment severe osteoporosis [18].

## 2. Experimental Section

### 2.1. Materials

Recombinant human growth hormone (rhGH) at a concentration of 13 mg/mL with 19 mM sodium phosphate buffer, 13.3 mg/mL glycine and 66 mg/mL mannitol was purchased from AMEGA Biotech (Buenos Aires, Argentina). rhGH activity was 3 IU/mg. The API (active pharmaceutical ingredient) solution was further processed prior to use as described in the Methods Section. Sucrose NF (National Formulary) (high purity, low endotoxin grade) was obtained from Pfanstiehl Laboratories (Waukegan, IL, USA). Polysorbate 20 (Crillet 1 HP, high purity, low peroxide) was sourced from Croda (Edison, NJ, USA). Titanium metal sheet (commercially pure grade 2, 25 μm in thickness) was obtained from Hamilton Precision Metals (Lancaster, PA, USA).

The ZP-patch system consists of a titanium array of 580 microneedles per cm^2^. The microneedles are 340 μm in length and 70 μm in width with tip angle of 60° (Kemac, Azusa, CA, USA). Other patch components include a polycarbonate ring (Jatco, Union City, CA, USA), adhesive patch (Adhesive Research Inc., Glen Rock, PA, USA), 3 Å molecular sieve desiccant co-molded into the polycarbonate ring (CSP Technologies, Auburn, AL, USA), and an aluminum foil pouch (Mangar, New Britain, PA, USA).

Norditropin^®^ (recombinant human growth hormone; 3.33 mg/mL) was produced by Novo Nordisk (Bagsvaerd, Denmark), purchased from a commercial source and stored per the manufacturer’s instructions.

### 2.2. Methods

#### 2.2.1. Diafiltration and Concentration

Diafiltrated and concentrated rhGH solution was prepared utilizing an Amicon Ultra-15 (Millipore, Billerica, MA, USA) centrifugal filter unit with a 10 kDa molecular weight cut off regenerated cellulose membrane. Approximately 15 mL of the rhGH solution was placed in the filter unit and centrifuged with a Beckman centrifuge model GS-15R (Beckman, Palo Alto, CA, USA) at RCF 2700*g* for 30 min. The solution was maintained at 4 °C during the centrifugation process. Following diafiltration the volume of the protein solution was reduced to a third of the original volume. Recovery of the rhGH concentrate from the Amicon Ultra-15 centrifugal filter units was high, typically >90% as determined UV spectroscopy. Sucrose was subsequently added to the concentrated rhGH solution at 1:1 ratio and then lyophilized.

#### 2.2.2. Lyophilization

Freeze drying was performed using a LyoStar II lyophilizer (FTS Systems, Stone Ridge, NY, USA) using the following cycle: freezing at −40 °C for two hours; primary drying for two hours under the vacuum of 350 mTorr at each temperature of −40, −30, −20, −10, and 0 °C; secondary drying at 10 °C/350 mTorr for 2 h, 20 °C/350 mTorr for 2 h, 30 °C/350 mTorr for 1 h, 30 °C/50 mTorr for 0.5 h, and 30 °C/0 mTorr for 0.5 h. Temperature was ramped up at 5 °C/min consistently.

#### 2.2.3. Size Exclusion Chromatography (SEC)

Soluble aggregates of rhGH were determined by the SEC-HPLC method at wavelength of 214 nm. Chromatography for the assay was performed using a Tosoh Bioscience TSKgel Super SW2000 column (4.6 mm ID × 300 mm, 5 µm) (Tosoh Bioscience, King of Prussia, PA, USA) with an isocratic mobile phase (97% phosphate buffer at pH 7.0 and 3% iso-propanol) at a flow rate of 0.2 mL/min, on an HPLC system (Water Alliance 2695, Waters Corporation, Milford, MA, USA) equipped with a binary pump, a thermostatted autosampler and column compartment, and a photodiode array detector (PDA). Data were collected and analyzed using Empower Pro (Empower 2 software, Waters Corporation).

#### 2.2.4. Recombinant Human Growth Hormone (rhGH) Purity Determination

Purity of hGH was determined by the reverse phase (RP)-HPLC method at wavelength of 220 nm. Chromatography for the assay was performed using an Agilent Zorbax 300SB-C8 column (4.6 mm ID × 150 mm, 3.5 µm) (Agilent, Santa Clara, CA, USA) with an isocratic mobile phase (71% Tris buffer at pH 7.5; 29% *n*-propanol) at a flow rate of 0.5 mL/min, on an HPLC system (Water Alliance 2695) equipped with a binary pump, a thermostatted autosampler and column compartment, and a PDA detector. Data were collected and analyzed using Empower Pro (Empower 2 software, Waters Corporation).

#### 2.2.5. rhGH Content Quantification

rhGH content was determined by the RP-HPLC method at wavelength of 220 nm. Chromatography for the assay was performed using a Agilent Zorbax 300SB-C8 column (4.6 mm ID × 50 mm, 3.5 µm) with an gradient mobile phase (45% of 0.1% trifluoroacetic acid (TFA) in water and 55% of 0.09%TFA in acetonitrile from 0 to 2 min, and a wash from 2 to 3 min) at a flow rate of 1.0 mL/min, on an HPLC system (Water Alliance 2695) equipped with a binary pump, a thermostatted autosampler and column compartment, and a PDA detector. Data were collected and analyzed using Empower Pro (Empower 2 software, Waters Corporation). Quantitation was done based on a four-point standard curve (typically 500, 750, 1000 and 1500 μg/mL) prepared from rhGH USP (United States Pharmacopeia) reference standard (Rockville, MD, USA) in Tris buffer at pH 7.5.

#### 2.2.6. Rheology

Rheological characterization of the liquid formulations was conducted utilizing a rheometer (model CVOR150, Bohlin Instrument, Cranbury, NJ, USA) configured with a cone and plate geometry (a cone angle of 1° and radius 10 mm). Seventy microliters of the liquid formulation was utilized for each experiment.

#### 2.2.7. Scanning Electron Microscopy (SEM)

Scanning electron microscopy (SEM) using a Hitachi scanning electron microscope (Model S-2460N) (Hitachi, Tokyo, Japan) was used to view the coating on the microneedles.

#### 2.2.8. Contact Angle Measurement

Static contact angle of drug solution formulations on titanium surface was determined using a Future Digital Scientific contact angle meter Model OCA15 (Garden City, NY, USA) employing the “Sessile drop” optical contact angle method. For static contact angle measurement, a photo snapshot was taken once a drop of the solution (5 μL) is dispensed from the syringe and laid on a clean titanium foil surface. The angle between the baseline of the drop and the tangent at the drop boundary is measured on both sides. Complete measurement was obtained by averaging the two numbers. A minimum of five readings were recorded for each sample.

#### 2.2.9. Microneedle Array Coating and Packaging

Titanium microneedle arrays were fabricated by a photo/chemical etching and formed using a controlled manufacturing process [19]. Drug formulation (20% *w*/*w* rhGH, 20% *w*/*w* sucrose and 0.2% *w*/*w* polysorbate 20) coating on the microneedle array was conducted at ambient temperature utilizing a roller drum, rotating at 50 rpm, in a drug formulation reservoir (2 mL in volume) to produce a drug formulation film of controlled thickness [20]. Microneedle tips on the array are dipped into the thin film and the coating per area controlled by the number of dips (passes) through the drug film. The time between each dip coating was less than 5 s, which was sufficient to dry the formulation coating.

The rhGH-coated microneedle array was assembled with adhesive and retainer ring (Figure 1a). The patch in retainer ring was packaged in an aluminum pouch (Mangar, New Britain, PA, USA) purged with dry nitrogen and heat-sealed with a Multivac heat sealer (model C400) (Multivac, Kansas City, MO, USA).

#### 2.2.10. Stability Experiments on Packaged Drug-Coated Microneedle Delivery Systems

The ZP-hGH patch in the sealed pouch was stored in a stability chamber (Model 6010, Caron, Marietta, OH, USA) controlled at 40 °C/60% RH (relative humidity). At each time point, three pouch samples were pulled from the stability chamber for HPLC analysis. To extract rhGH from the coated array, the array was first separated from the adhesive by exposing to liquid nitrogen vapor and then peeled from the adhesive. The coated array was then placed in a vial containing 0.5 mL of sodium phosphate buffer (pH 7.4) and mixed for a period of 5 min, of which 250 μL of the resulting solution was transferred into a secondary vial for SEC and RP-HPLC analysis.

### 2.3. Preclinical Pharmacokinetic Studies

#### 2.3.1. Animal Model and Delivery Preparation

Female hairless guinea pigs (HGP) (body weights 0.4–0.5 kg) were obtained from Charles River Laboratories (Wilmington, MA, USA). All animal studies adhered to the NIH Principles of Laboratory Animal Care [21] and were in compliance with the animal welfare regulations in 9 CFR 1–3, the National Research Council Guide for the Care and Use of Laboratory Animals 1996 [22] and an approved institutional animal care and use committee protocol.

HGPs were anesthetized by intramuscular injection of xylazine (8 mg/kg) and ketamine HCl (44 mg/kg). To minimize the stress of anesthesia, the animals were kept warm on a circulating water pad at 37 °C and carefully monitored. Anesthetized HGPs were catheterized in the carotid artery. The catheter was flushed with saline. Animals were maintained under anesthesia throughout the experiment via injection of sodium pentobarbital (32 mg/mL) directly into the catheter (0.1 mL/injection).

#### 2.3.2. Zosano Pharma (ZP)-hGH Patch Application, Sub-Cutaneous (SC) and Intravenous (IV) Dosing

ZP-hGH patches were applied to the lateral thorax of HGP (*N* = 5) using a hand held re-usable applicator (total energy = 0.29 J). The patch was attached to the applicator and the applicator was pressed on the skin, releasing the patch and applying it with a predetermined force using a previously described method [23]. ZP-hGH patches were applied and worn for 1 h then removed. The ZP-hGH patch doses tested were; 0.5 and 1 mg. For the 1 mg dose, two 0.5 mg patches were applied.

Norditropin^®^ SC injections of 0.4, 0.8 and 1.4 mg were used as comparators. Injections were made on the lateral thorax consistent with the patch application sites.

ZP-hGH patch delivery efficiency was assessed by residual drug analysis. The amount of rhGH left on the microneedle array and skin surface after each application were compared against the original coated amount on the microneedle array.

Intravenous dosing of rhGH was used to evaluate the absolute bioavailability of Sub-Cutaneous (SC) injections and rhGH coated patches. The left or right jugular vein of the anesthetized HGP was catheterized (after carotid catheterization). rhGH injectate was formulated at 205 μg/mL in saline and administered at a dose of 105 μg/kg body weight via direct injection through the jugular catheter. A 0.1 mL dead volume of the catheter was accounted for in the dosing. The rhGH injectate administered intravenously to all animals were ≤0.25 mL and based on a dose volume 0.5 mL/kg body weight.

Local skin tolerability of the ZP-hGH patch was evaluated using the Draize scoring system [24]. Skin site evaluations were made immediately following patch removal.

#### 2.3.3. Pharmacokinetic Studies (PK)

Pharmacokinetic samples were collected at 0, 5, 15, 30, 60, 120, 180, and 240 min to evaluate systemic absorption of rhGH. At each time point, a 0.2 mL blood sample was collected from carotid artery. Whole blood samples were centrifuged (4000 rpm, 8 min, 4 °C), plasma collected and frozen (−80 °C) until assayed. Plasma rhGH was determined by enzyme immunoassay using a human growth hormone Quantikine ELISA kit obtained from the R&D Systems (Minneapolis, MN, USA). The sensitivity of the assay is 7.2 pg/mL with a coefficient of variation of <10%. The rhGH dose delivered by the patch was extrapolated based on the area under the curve (AUC) calculation compared to the SC injection control.

Plasma samples were obtained from alternating groups of animals to minimize the effect of sampling with of 5 animals per time point. Pharmacokinetic parameters were determined by non-compartmental analysis.

#### 2.3.4. Statistical Analysis

Results are presented as the mean ± SE. One way ANOVA was conducted to compare the means *p* < 0.05 was considered significant.

## 3. Results and Discussion

### 3.1. Formulation Characterization, Microneedle Coating and Stability

There are two processes, as defined by the coating operation, to produce the drug-coated patch dip coating of microneedles into a high concentration liquid drug formulation, as well as drying and packaging of the solid-state drug formulation, which have been discussed previously [25]. Briefly, a liquid formulation is prepared to primarily satisfy three key coating formulation parameters: drug concentration, viscosity, and surface activity.

**Figure 2:**
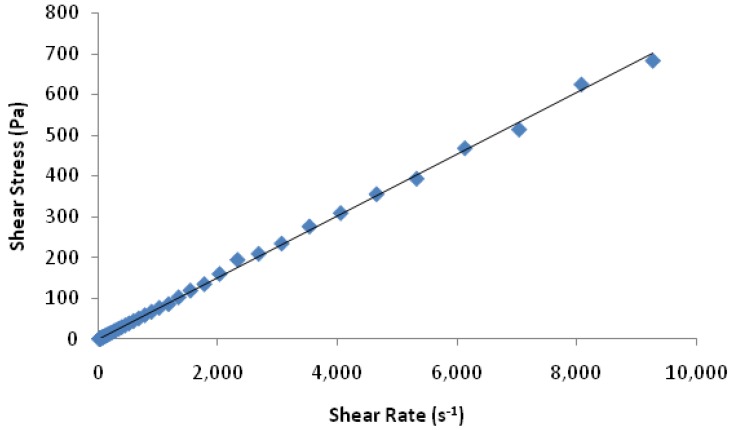
Shear stress *vs.* shear rate for 20% *w*/*w* rhGH, 20% *w*/*w* sucrose, 0.2% *w*/*w* polysorbate 20 liquid formulation. Shear stress is proportional to shear rate suggesting Newtonian behavior for the rhGH liquid formulation.

On this basis a liquid 20% *w*/*w* rhGH, 20% *w*/*w* sucrose and 0.2% *w*/*w* polysorbate 20 was prepared. Arriving at this formulation composition was partially driven by chemical stability considerations. The rhGH liquid formulation was formulated to a high concentration (200 mg/mL; 20% *w*/*w*) to ensure that each dip of microneedles into the liquid formulation can pick up sufficient volume of liquid for drying, which can achieve the desired drug dose with a minimum number of dips. In addition the viscosity of the coating solution, 74 ± 2 cP, was high enough so that the coated liquid will not quickly drip back after dipping but before drying. Sucrose was the primary protein stabilizer and its content was limited to a 1:1 sucrose: rhGH *w*/*w* ratio because increasing sucrose content would add more solid to the coating of the same rhGH dose on the microneedle tips which would eventually blunt the sharpness and hinder skin penetration. A surfactant, polysorbate 20, (0.2%) was added to the liquid rhGH formulation. This served two purposes firstly as a stabilizer to reduce the formation of insoluble protein aggregates [26] and to decrease the contact angle to 50° and improve the wetting and coating on the titanium microneedle surface. The rhGH liquid formulation exhibited Newtonian behavior (Figure 2), with the shear stress proportional to the shear rate. This formulation property facilitates a uniform coating of the microneedles as described previously [20].

The rhGH liquid formulation was then coated on titanium microneedle array. Figure 1c shows the uniform formulation coating on each of the microneedles within the array as demonstrated by scanning electron microscopy (SEM). The rhGH coating is observed on the microneedle and not on the array structure. The localization of the coating on the microneedle (Figure 1d) is consistent with the thickness of the drug film established by the roller drum. The coating surface is smooth. The rhGH coated amount per square centimeter was 167 ± 2.4 μg and was consistent from batch to batch.

**Figure 3:**
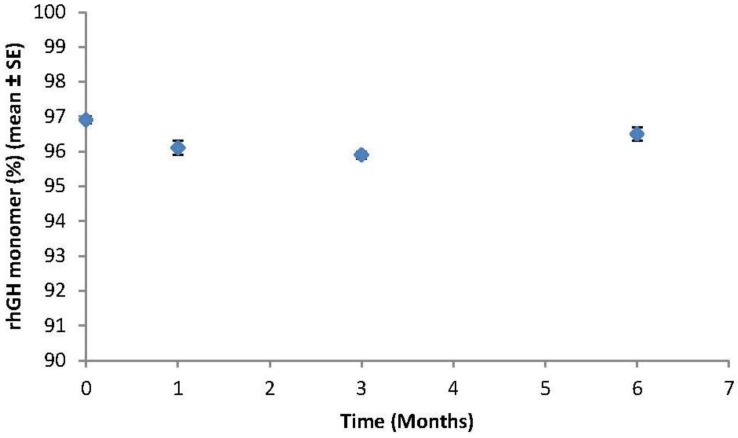
Stability of rhGH-coated microneedle system where the 3 cm^2^ titanium microneedle array was coated with 0.5 mg dose, assembled on a 5 cm^2^ adhesive patch within a polycarbonate retainer ring with co-molded desiccant, and heat sealed in a nitrogen-purged foil pouch. The final rhGH-coated microneedle packaged systems were stored at 40 °C/75% RH (relative humidity).

**Figure 4:**
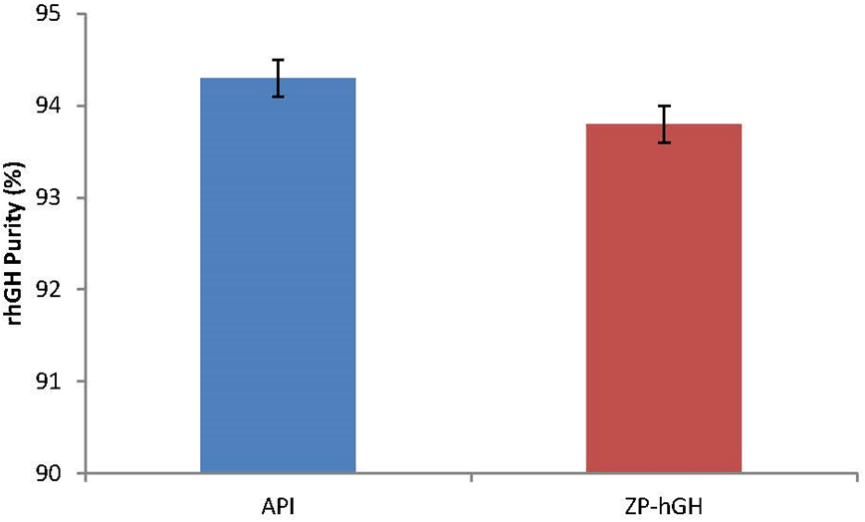
Comparison of the purity of rhGH active pharmaceutical ingredient (API) prior to processing and ZP-hGH patch after storage at 40 °C/75% RH for 6 months.

The assembled ZP-hGH microneedle patches (Figure 1a) were stored at 40 °C/75% RH. Figure 3 summarizes rhGH monomer (%) data for 40 °C/75% RH up to 6 months. Within the time course of the study ZP-hGH systems held excellent stability; rhGH % monomer at the 6-month time point is similar to that at *T* = 0 and showed no obvious trend of decreasing. The purity of the rhGH coated on the patch was also evaluated and compared with the API. Figure 4 shows that after 6 month storage at 40 °C/75% RH there was no difference in the purity of the rhGH coated on the patch relative to the API or rhGH coated patch at *T* = 0.

### 3.2. rhGH-Coated Microneedle Delivery Performance and Release Kinetics

**Figure 5:**
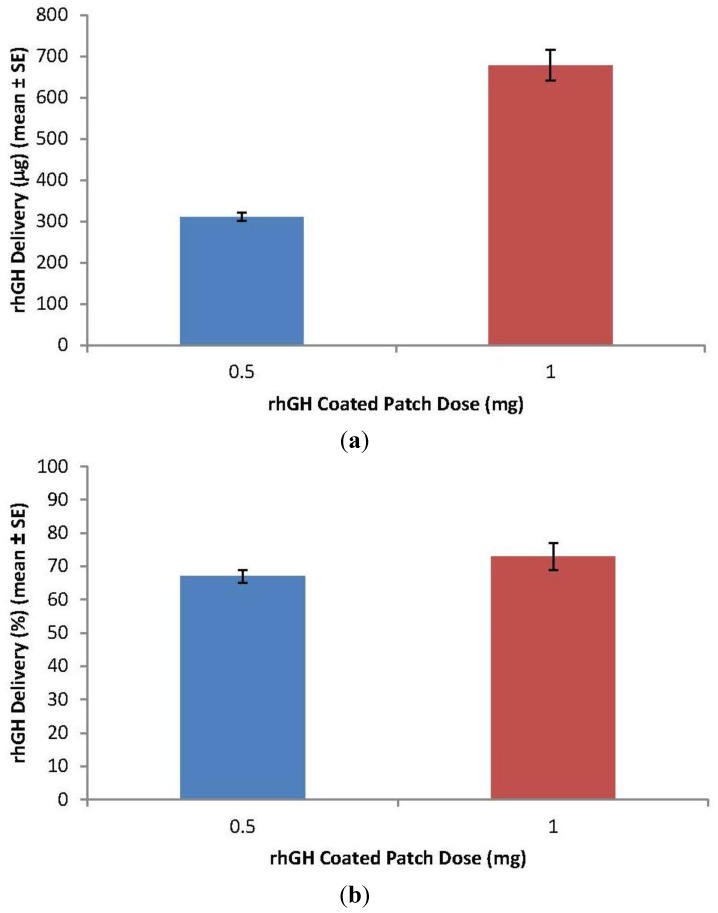
ZP-hGH delivery performance. (**a**) delivery into HGP skin; (**b**) delivery efficiency (%) with ZP-hGH patch wear time of 1 h. Patches were tested at doses of 0.5 and 1 mg.

rhGH delivery into skin with a patch wear time of 1 h was: 334 ± 8 and 678 ± 26 μg (mean ± SE) for rhGH-coated doses of 0.5 and 1 mg, respectively. Delivery efficiency was excellent with *ca.* 70% of the rhGH-coated dose delivered into the skin (Figure 5a,b).

Local skin tolerability was rated as very good. The erythema scores (maximum possible score: 4) [20] never exceeded 2 and were predominately 0–1 representing no or barely perceptible erythema. There was no evidence of topical bleeding with any of the treatment conditions. Primary irritation indices (PII) (maximum possible score: 8) were 0 to 0.5 and thus categorized as negligible to slight.

### 3.3. Pharmacokinetic Data

**Figure 6:**
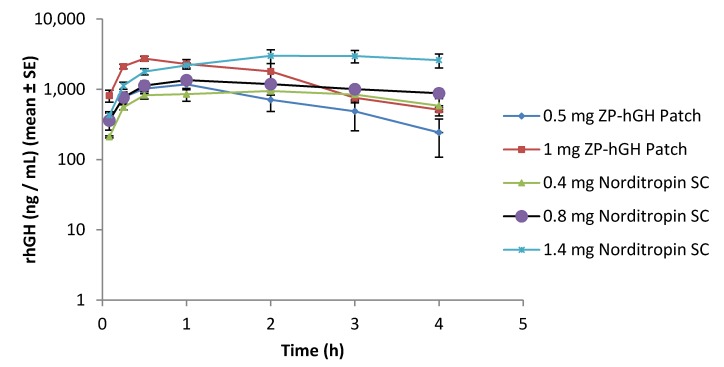
Pharmacokinetic (PK) profile of rhGH delivered by microneedle patch or SC injection: ZP-hGH patch (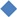 0.5 mg and 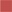 1 mg); Norditropin^®^ SC injection 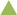 0.4 mg, 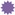 0.8 mg and 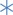 1.4 mg).

**Figure 7:**
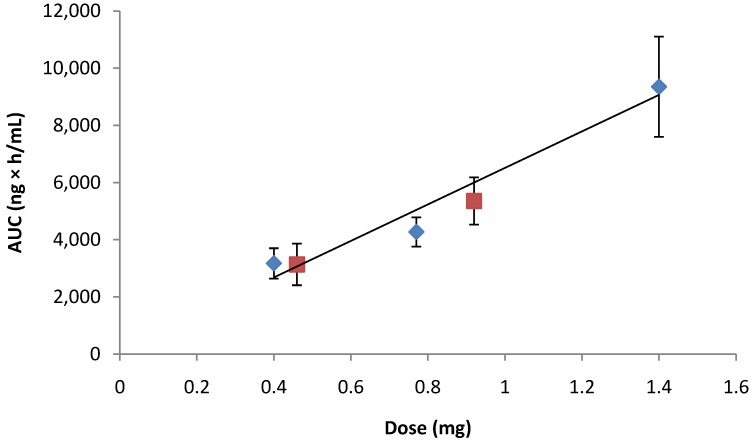
rhGH plasma concentration of ZP-hGH patch at 0.5 mg and 1 mg doses (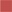) *vs.* Norditropin^®^ SC injection over the range of 0.4 to 1.4 mg (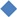).

Preclinical *in vivo* evaluation showed rapid systemic absorption of rhGH with patch administration (Figure 6). There was a linear dose response in terms of both area under the curve (*AUC_t_*) and maximum observed plasma concentration (*C*_max_) (Figure 7).

Plasma levels were maintained over 4 h with *T*_max_ at 30 min and a plasma half-life of 70 min (Table 1). rhGH patches had an absolute bioavailability of 25%, similar to SC injected Norditropin^®^. The *C*_max_ of the 0.5 mg rhGH patch was similar to the 0.8 mg SC injection. The calculated elimination rate constant (*K*_el_) for the patch was 0.5 h^−1^ compared to 0.15 h^−1^ for the SC injection. It is not clear from this study whether the administered human doses in this animal model will translate to a faster or equivalent PK profile in the clinic. Other studies suggest that drug-coated microneedle patch delivery can produce a rapid-on and rapid-off PK profile [23,27,28]. This type of delivery may have an advantage over SC administration as growth hormone is secreted in a pulsatile manner in humans [29,30]. In addition, clinical trials suggest that pulsatile growth hormone may be required to increase lipolysis [31,32], while non-pulsatile circulating growth hormone levels may adversely affect insulin sensitivity [33]. However, despite the presumed benefit of pulsatile delivery, recent studies suggest that a long-acting rhGH may be effective in growth-hormone-deficient adults as measured by IGF-1 responses [34]. Nevertheless, a drug-coated microneedle patch still may be an advantageous route of administration for either form of rhGH and provide patients an alternative to needle sticks over the prolonged treatment duration.

**Table 1:** Comparison of PK parameters for intravenous (IV), ZP-hGH and Norditropin^®^ SC Injection.

PK parameters	IV	ZP-hGH		SC Norditropin		
Dose (mg)	0.05	0.5	1	0.4	0.8	1.4
*C*_max_ (ng/mL)	4,766 ± 421	1,185 ± 207	2,799 ± 223	967 ± 133	1,418 ± 187	3,193 ± 613
*T*_max_ (min)	1	30	30	120	60	120
*T*_1/2_ (min)	25 ± 2.5	67 ± 13	73 ± 12	151 ± 38	291 ± 34	362 ± 19
*AUC_t_* (ng × h/mL)	1,105 ± 145	2,774 ± 730	5,352 ± 924	3,171 ± 530	4,270 ± 513	9,352 ± 1753
Absolute Bioavailability (%) *	Reference	27 ± 6	26 ± 4	36 ± 5	24 ± 2	30 ± 4

*AUC_t_* (*t* = 0–4 h), * presented using calculation with dose normalization.

## 4. Conclusions

These studies demonstrate for the first time that a stable, therapeutically relevant rhGH doses can be administered from a microneedle patch with high efficiency and bioavailability.

The rhGH can be formulated at high concentration and coated onto a microneedle patch without generation of soluble aggregates. The rhGH coating formulation at ~200 mg/mL allowed uniform and localized microneedle coating. ZP-hGH patches stored in sealed nitrogen purged foil pouches with desiccant were stable at 40 °C storage for at least six months with no significant changes in aggregation and purity.

Preclinical delivery studies in a HGP model showed high drug delivery efficiency for the ZP-hGH patches and a linear dose response at therapeutically relevant SC doses for Norditropin^®^. The bioavailability of the rhGH microneedle patch was similar to the comparable subcutaneous injected Norditropin^®^ dose. These results suggest that transdermal microneedle patch delivery of rhGH is feasible and warrants further development as a patient-friendly alternative to current subcutaneous injection products.
